# Laser stricturotomy-assisted rendezvous ERCP and cholangioscopy for post-cholecystectomy complete common bile duct transection

**DOI:** 10.1055/a-2743-3956

**Published:** 2025-11-27

**Authors:** Sharan Malipatil, Jaydeesh Khanna Balasubramanian, Nagesh Kamat, Sanil Parekh, Biswa Ranjan Patra, Sehajad Vora, Amit Maydeo

**Affiliations:** 1 81727 Sir HN Reliance Foundation Hospital and Research Centre, Institute of Gastrosciences, Mumbai, India; 2 150819 Khoo Teck Puat Hospital, Singapore

Complete transection of the common bile duct (CBD) during cholecystectomy is a serious injury 1 that can lead to significant postoperative morbidity requiring prompt management 2 . Endoscopic retrograde cholangiopancreatography (ERCP) alone is often insufficient due to the inability to bridge the disconnected duct ends 3 . We used a novel technique to bridge the transected bile duct ( Video 1 ).

Rendezvous ERCP with thulium laser stricturotomy and cholangioscopy to bridge the transected bile duct.Video 1

**Fig. 1:**
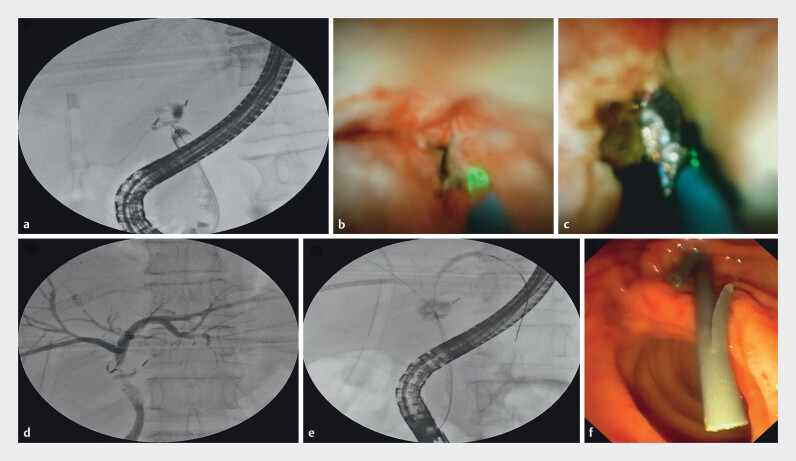
**a** Cholangiogram showing complete transection of the CBD (Strasberg E2). **b** Stricturotomy with thulium laser. **c** Bile trickling out through ablation site, surgical clips noted in situ. **d** PTBD cholangiogram showed intrahepatic biliary radicles with a thin rim of contrast entering the CBD across the transected site. **e** Guidewire introduced into the right and left hepatic duct. **f** Placement of plastic stents in left and right hepatic duct.

A 65-year woman, post laparoscopic cholecystectomy, was referred for bile leak with a percutaneously placed drain. Magnetic resonance cholangiopancreatography suggested complete transection (~ 2 cm) of the proximal CBD just below the hilar confluence (Strasberg E2 bile duct injury). During ERCP, a guidewire (Terumo 0.032" angled tip) could not be passed across the transection ( Fig. 1 **a** ). We introduced a SpyGlass cholangioscope (Boston Scientific, United States), which showed a stricture at the CHD level. A thulium laser probe (Electro Medical Systems, Switzerland) was introduced through the cholangioscope ( Fig. 1 **b** ) and the fibrotic stricture was ablated (tissue ablation - energy 1J, frequency 10 Hz, power 10W, medium pulse length), gaining access to the collection, which visualized the surgical clips. Bile trickled through the ablation site ( Fig. 1 **c** ), but we could not pass the cholangioscope/guidewire across the stricture due to an acute angle. EUS-guided antegrade biliary drainage was attempted, but it was not feasible due to a non-dilated duct and excessive breathing movements. A cholangiogram through percutaneous transhepatic biliary drainage (PTBD) site was obtained using a NEFF percutaneous access set. Mild intrahepatic biliary dilatation was seen, with a leak from the hilum into the drain with no opacification of the CBD. The right anterior duct was punctured, and an 8F external drainage catheter was inserted across the hilum. A cholangiogram done through the PTBD site ( Fig. 1 **d** ) showed a dilated right and left biliary radicle with a thin rim of contrast entering the CBD across the transected site. Using the rendezvous technique, a guidewire (Terumo) was passed in antegrade fashion from the PTBD site into the CBD. The guidewire was pushed across the ampulla and retrieved into the scope using rat tooth forceps (Olympus, United States). Keeping the guidewire in the right hepatic duct, another guidewire was manipulated with difficulty and placed in the left hepatic duct (12 cm) ( Fig. 1 **e** ). Two 7F plastic stents were placed in the left hepatic duct (10 cm) and right hepatic duct (12 cm) ( Fig. 1 **f** ). At discharge, the biliary drain output had stopped. At 3-month follow-up, ERCP confirmed no bile leak, indicating successful management. This procedure restored biliary continuity, reduced hospitalization time, and avoided complex surgery.
